# All-fibre phase filters with 1-GHz resolution for high-speed passive optical logic processing

**DOI:** 10.1038/s41467-023-37472-2

**Published:** 2023-03-31

**Authors:** Saket Kaushal, A. Aadhi, Anthony Roberge, Roberto Morandotti, Raman Kashyap, José Azaña

**Affiliations:** 1 Énergie, Matériaux et Télécommunications, Institut National de la Recherche Scientifique, 1650 Lionel-Boulet Blvd., Varennes, J3X 1P7 Quebec Canada; 2 Department of Engineering Physics, Fabulas Laboratory, Polytechnique Montréal, 2500 Chem. de Polytechnique, Montréal, H3T 1J4 Quebec Canada; 3 Department of Electrical Engineering, Fabulas Laboratory, Polytechnique Montréal, 2500 Chem. de Polytechnique, Montréal, H3T 1J4 Quebec Canada

**Keywords:** Fibre optics and optical communications, Integrated optics

## Abstract

Photonic-based implementation of advanced computing tasks is a potential alternative to mitigate the bandwidth limitations of electronics. Despite the inherent advantage of a large bandwidth, photonic systems are generally bulky and power-hungry. In this respect, all-pass spectral phase filters enable simultaneous ultrahigh speed operation and minimal power consumption for a wide range of signal processing functionalities. Yet, phase filters offering GHz to sub-GHz frequency resolution in practical, integrated platforms have remained elusive. We report a fibre Bragg grating-based phase filter with a record frequency resolution of 1 GHz, at least 10× improvement compared to a conventional optical waveshaper. The all-fibre phase filter is employed to experimentally realize high-speed fully passive NOT and XNOR logic operations. We demonstrate inversion of a 45-Gbps 127-bit random sequence with an energy consumption of ~34 fJ/bit, and XNOR logic at a bit rate of 10.25 Gbps consuming ~425 fJ/bit. The scalable implementation of phase filters provides a promising path towards widespread deployment of compact, low-energy-consuming signal processors.

## Abstract (web summary)

Authors present a fibre Bragg grating-based all-pass spectral phase filter with an unprecedented frequency resolution of 1 GHz, at least 10× improvement compared to a standard optical waveshaper. Using the all-fibre phase filter, fully passive NOT and XNOR logic operations are experimentally demonstrated at ultrafast speeds with few-fJ/bit energy consumption.

## Introduction

The generation, processing, detection, and analysis of high-speed temporal signals^1–4^ underpin a wide range of information and communication technology (ICT) applications. Electronic technologies have been very successful in providing the needed solutions for realization of these tasks, mainly driven by the digital signal processing (DSP) framework^5,6^. However, these technologies suffer from fundamental limitations and trade-offs regarding their processing speed or bandwidth capabilities and their energy consumption^7–9^. The latest represents an increasingly significant problem. For instance, it is predicted that the energy consumption of the ICT sector alone would account for at least 20% of the global total by the end of 2030^10^, mainly due to the expected continuous growth of internet traffic and all related services^11^. In this context, by harnessing the remarkable frequency bandwidth of light waves (well above the THz range), photonic technologies have shown great promise for the realization of many of the needed temporal signal generation, processing, and analysis functionalities at speeds orders of magnitude higher than those enabled by electronic solutions^12–14^. However, compared to electronic devices and systems, photonic solutions remain power-hungry^15^, a crucial drawback for their use in real-world settings. Towards efficient and high-speed signal processing, photonic schemes based on all-pass (or phase-only) linear spectral filtering have attracted considerable recent interest. These schemes offer a simple and passive solution to realize a multitude of important functionalities, such as ultrafast logic gates^16^, ultrahigh-speed spectroscopy^17^, quantum states manipulation^18,19^, microwave photonics operations^20^, neuromorphic computing^13^, pulse shaping and arbitrary waveform generation^21,22^, among many others. The key advantage of linear spectral phase-only optical filtering is that it enables implementation of the desired functionality at speeds commensurate with a photonic solution while preserving the full energy of the processed temporal signal. In practice, the energy consumption is just limited by the practical insertion loss of the phase-only filter, which can be optimized to be particularly low. This methodology has enabled remarkable improvements in energy efficiency as compared to photonic processing schemes based upon either nonlinear optics or even linear arbitrary (generally, amplitude-dependent) optical filtering^23,24^.

In spite of the tremendous potential of the photonic phase filtering approach, implementations of the resulting schemes in practical compact and fully passive formats, such as in fibre-optics or integrated waveguide technologies, have remained elusive. In most demonstrations, a waveshaper based on free-space diffraction gratings and liquid-crystal-on-silicon (LCOS) technology^25^ has been used to provide the required optical phase filtering functionality. Such a waveshaper, although programmable, is inherently bulky, requires ~40 W power for operation, and offers limited frequency resolution (~13 GHz). The latest severely restricts the performance and range of operation of the demonstrated processors, e.g., in terms of minimum frequency spacing for manipulation of optical combs^21,26^ or the maximum temporal duration of the output waveforms in pulse shaping or arbitrary waveform generation^27^. In this regard, line-by-line pulse shapers employing a waveguide grating router (WGR)^28^ or virtual imaged phase arrays (VIPAs)^29^ as diffractive elements have been demonstrated to realize improved spectral resolution metrics, albeit over a finite free spectral range (FSR). Yet, all the proposed solutions remain bulky and require additional free-space components. Thus, it is imperative to look for a scalable and integrated technology solution that has the potential to implement these novel functionalities in a practical, compact and fully passive platform and with improved performance. In this respect, fibre Bragg gratings (FBGs)^30^, or their integrated-waveguide counterparts^31^, represent a particularly promising alternative. An FBG is created through a well-designed quasi-periodic perturbation of the effective refractive index along the length of an optical fibre, inducing a user-defined coupling between a forward and a backward propagating fibre mode. This technology has enabled many important applications in fibre-optics telecommunications and in a wide range of other fields. The unique advantage of FBGs is that they can be designed to provide a fully customized linear spectral response (both in amplitude and phase) by suitable apodization of the coupling coefficient along the length of the grating while operating in reflection. However, to realize GHz to sub-GHz resolution in either the grating amplitude or phase response requires ultra-long FBGs, with device lengths typically > 15 cm^32^. It is difficult to precisely define and control the amplitude and phase of the coupling coefficient in ultra-long FBGs owing to non-uniformity in the fibre’s parameters^33^, which leads to significant phase errors, thus degrading the spectral response^34,35^. Optical filters with a well-defined amplitude spectral response (e.g., a narrow pass band) have been designed with resolutions below the GHz range^36^. However, it is much more challenging to control the filter’s phase response, particularly in an all-pass filtering device. As such, the problem of realizing a customized phase filtering functionality with GHz to sub-GHz resolution in an integrated device remains largely unsolved.

Towards this aim, we demonstrate an FBG-based all-pass spectral filter providing a target phase response with an unprecedented frequency resolution of 1 GHz. This represents an improvement of >10× over the frequency resolution of an optical waveshaper, and moreover, this is achieved using a compact and passive fibre-optics device. To this end, a phase modulation-based apodization scheme is employed to realize the high dynamic range of the required coupling coefficient profile of the advanced FBG structure. The FBG is fabricated using a femtosecond laser-based plane-by-plane direct-writing scheme^37^. By leveraging the superior spatial resolution offered by the femtosecond laser compared to a traditional UV-based interferometric writing scheme, a precise control of the coupling coefficient over long lengths ( > 21 cm) is feasible. To showcase the unique potential of the fabricated phase filter, we have harnessed its unparalleled frequency resolution to experimentally demonstrate an all-passive, high-speed implementation of two fundamental building blocks in computing circuits and information processing — NOT and XNOR logic gates. Electronic transistors-based logic gates consume a significant amount of energy (~8.6 pJ/bit), even at speeds just above several Gigabits per second (Gbps)^38^. In this aspect, photonic-based implementations of logic gates have been proposed by exploiting either nonlinear optics effects^39^ or through linear interferometry using a control beam^40^. However, all the current alternative techniques fail to provide the required performance, compared to electronic logic gates, simultaneously in three important aspects: energy consumption, footprint, and operation speed. Passive optical logic gate designs based on spectral phase filtering have been demonstrated that enable ultrafast operation with potential minimal power consumption^16^, but their previous implementations still necessitate of a bulky and active waveshaper.

In this work, we report through proof-of-concept experiments, linear, fully passive, high-speed NOT and XNOR logic gates for data bits encoded in the non-return to zero on-off keying (NRZ-OOK) format. Particularly, we demonstrate an all-fibre realization of the NOT logic at a bit rate of 45 Gbps with an estimated energy consumption of ~34 fJ/bit. The superior frequency resolution of the FBG is the key in enabling efficient operation of the logic gates over a much wider range of bit rates, i.e., down to tens of Gbps, compared to previously reported demonstrations wherein the limited frequency resolution of a waveshaper severely restricted the minimum signal bit rate to 640 Gbps^16^. The FBG has also been utilized to experimentally realize a fully passive XNOR logic at a bit rate of 10.25 Gbps with an estimated energy consumption of ~425 fJ/bit. The demonstrated passive logic can be exploited to realize many important computing applications^41^, such as a pattern matching technique proposed here, with a potential to enable significantly faster processing speeds with reduced power consumption compared to state-of-the-art search algorithms. The remarkable advantage offered by the demonstrated in-fibre processors in regard to size, weight, power, and cost (SWaP-C) could enable the realization of many other all-optical signal processing functionalities, paving the path towards a widespread application of photonic-based high-speed signal processing devices and systems with optimal efficiency and reduced footprint.

## Results

### FBG-based phase filter: design and characterization

**Fig. 1:**
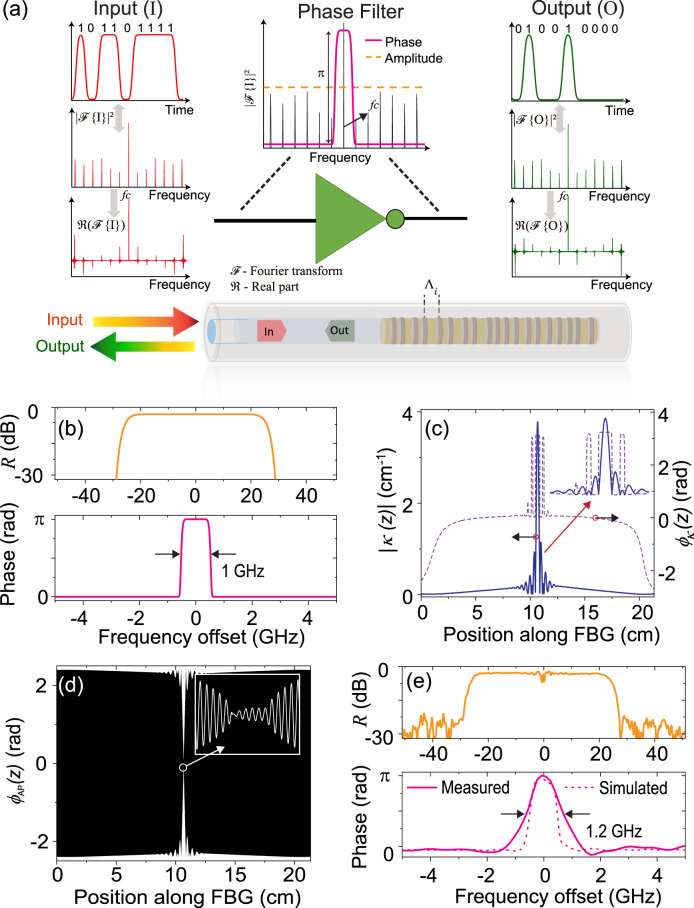
A fibre Bragg grating-based linear passive NOT gate for high-speed NRZ-OOK signals. **a** A selective application of a *π* − phase shift on the central or carrier frequency component, *f*_*c*_ ensures inversion of the entire input data stream at once in the time-domain^16^. This is possible as the spectrum ($|{{{{{{{\mathscr{F}}}}}}}}\,(\cdot ){|}^{2}$) of both the input and the output-inverted data streams are identical, except for the reversal in the phase of the individual spectral components corresponding to the data bits, as seen in the real part of the spectrum, $\Re ({{{{{{{\mathscr{F}}}}}}}}\,(\cdot ))$. A fibre Bragg grating (FBG), operating in reflection, is designed to realize the phase-only filter. The target response is realized by varying the separation, Λ_*i*_ between individual grating planes along the FBG. **b** Targeted reflectivity, *R* (top), and the spectral phase profile (bottom) of the FBG. **c** Required coupling coefficient profile *κ*(*z*) = ∣*κ*(*z*)∣*exp*(*jϕ*_*κ*_(*z*)) extracted by means of an inverse layer peeling algorithm. Inset shows the sharp oscillations in both the amplitude, ∣*κ*(*z*)∣ (solid) and phase, *ϕ*_*κ*_(*z*) (dashed) of the required coupling coefficient around the centre of the FBG. A slowly varying function, *ϕ*_AP_(*z*), is introduced in the phase function of the FBG to realize the required coupling coefficient profile, while maintaining a constant amplitude of the refractive index modulation throughout the FBG’s length. **d** Variation of *ϕ*_AP_ along the FBG’s length. Inset shows the zoomed-in periodic nature of *ϕ*_AP_. **e** Measured reflectivity (top) and associated spectral phase response along the passband (bottom) of the phase filter. The passband of the FBG is centred at ~ 1550 nm. The simulated phase response of the designed FBG is shown with a dotted trace.

The basic operation principle of the passive NOT gate for NRZ-OOK data signals is based on selectively imparting a *π* − phase shift to the central or carrier frequency component with respect to the continuous and much-weaker spectral components corresponding to the data bits^16^, as shown in Fig. 1a. This leads to a bit-by-bit inversion of the input data sequence. This design is inherently bit rate independent, as a single *π* − phase shift on the carrier frequency component is required for bit inversion. To implement this phase filter, an FBG operating in reflection is designed where the target spectral phase response is a rectangular (0 − *π*) phase shift, with a 3-dB bandwidth (BW) of 1 GHz, see Fig. 1b. We highlight that this specific spectral phase response is particularly difficult to be realized in practice, as two exact *π* − phase shifts should be implemented, up and down, respectively, over a very narrow bandwidth (1 GHz). The 3-dB BW of the target amplitude spectral response (nearly square shape) is 50 GHz, with a peak reflectivity of 50%. An inverse layer peeling algorithm^42^ is used to calculate the target coupling coefficient profile, *κ*(*z*) = ∣*κ*(*z*)∣*exp*(*jϕ*_*κ*_(*z*)), shown in Fig. 1c. As can be inferred from the profile, a high dynamic range and precise control of the coupling coefficient (in both its amplitude and phase) is required. We propose using a phase modulation of the grating structure to achieve the defined stringent phase filtering response^43^. Briefly, the target coupling apodization is achieved by incorporating a slowly varying sinusoidal phase component *ϕ*_AP_(*z*), (see Fig. 1d) in the phase function of the FBG, described by Eqn. (1) below,

1 $${n}_{{{{{{{{\rm{eff}}}}}}}}}(\lambda,\, z)={n}_{{{{{{{{\rm{eff}}}}}}}}}(\lambda )+\Delta n\cdot cos\left\{\frac{2\pi }{\Lambda }z+{\phi }_{{{{{{{{\rm{AP}}}}}}}}}(z)+{\phi }_{\kappa }(z)\right\}$$

Here *n*_eff_(*λ*, *z*) is the effective index profile of the FBG as a function of wavelength *λ* and device length *z*, Δ*n* is the amplitude of the constant refractive index modulation, Λ is the nominal grating period, *ϕ*_*κ*_(*z*) is the grating phase (or phase of *κ*(*z*)). A femtosecond laser based plane-by-plane direct-writing scheme is used for the inscription of the designed FBG^37^. A microcontroller-based resistive heating mechanism is utilized to locally correct for any fabrication imperfections-induced perturbations in the local Bragg wavelength along the FBG’s length. A detailed description of the involved FBG design and fabrication process is provided in the Methods section. The total length of the designed FBG is ~21.3 cm. The full complex spectral response of the fabricated FBG is measured using an optical vector analyzer (see Fig. 1e). As observed, the measured spectral phase response closely matches the designed phase response, with a 3-dB BW of the *π* phase-shifted frequency band (centred at ~1550 nm) of 1.2 GHz and a rectangular shape factor of ~0.54, where the shape factor is defined as the ratio between the 3-dB BW and the 10-dB BW of the *π* − phase shift^36^. The amplitude response exhibits a nearly square response with a peak reflectivity of ~45% (designed: 50%).

### Experimental demonstration of NOT gate

**Fig. 2:**
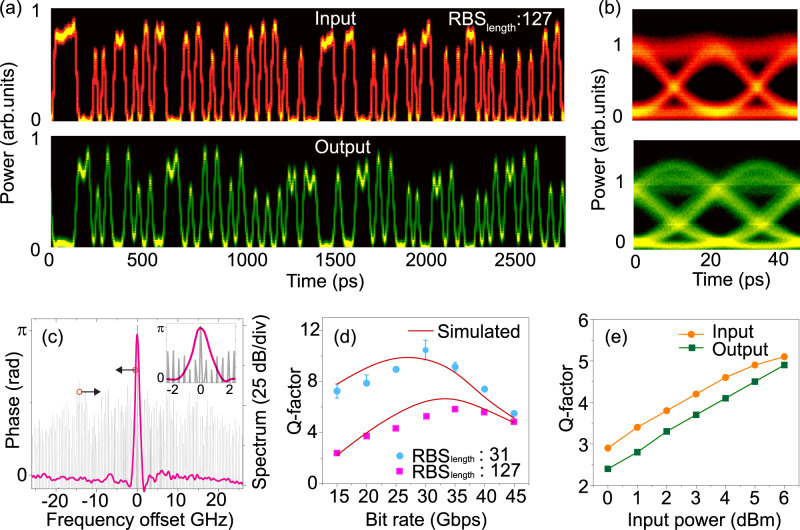
Experimental results related to NOT operation of NRZ-OOK signals using the phase filter. **a** Measured temporal waveforms at the input (top) and the output (bottom) of the phase filter. The input is a 45-Gbps NRZ-OOK signal with a random bit sequence (RBS) of 127 bits, centred at ~1550 nm wavelength. Temporal waveforms are recorded using an optical sampling oscilloscope (OSO). **b** Corresponding eye diagrams over a single bit period. **c** Measured spectral phase response of the phase filter superimposed with the measured spectrum of the input 45-Gbps NRZ-OOK, 127-bit RBS signal. The inset shows the zoomed-in *π* − phase shift of the phase filter around the carrier frequency of the input signal. The performance of the phase filter is evaluated using the Q-factor as a metric, where the Q-factor is calculated from the measured eye diagram. **d** Variation of the Q-factor of the output signal for different bit rates and RBS lengths of 31 bits (blue) and 127 bits (magenta). Measurements are recorded at a constant average power of 6 dBm. The error bars represent the maximum variation from the mean. **e** Sensitivity measurement of the OSO employing a 45-Gbps NRZ-OOK 127-bit RBS, for both input (orange) and output (green).

Figure 2a, b shows the measured temporal waveforms of the 45-Gbps 127-bit long random bit sequence (RBS) at the input (top) and the output (bottom) of the phase filter and the corresponding eye diagrams. Inversion of individual data bits is clearly evident, with negligible distortions on the temporal shape of the individual bits. We reiterate that the observed inversion is exclusively due to the application of a *π* − phase shift from the phase filter on the carrier frequency component of the input signal, see Fig. 2c. To ensure distortion-free inversion, the 3-dB BW of the (0 − *π*) phase shift should be less than, 2*B*/RBS_length_ where RBS_length_ is the length of the input RBS with bit rate *B*. A detailed derivation is presented in the Methods section. In order to quantify the performance of the NOT operation over different bit rates and RBS lengths, we calculated the corresponding Q-factor from the measured eye diagram of the output-inverted data signal. Here, Q-factor is the difference of the mean values of the two signal levels (a ‘0’ and a ‘1’ bit) divided by the sum of the noise standard deviations of the two levels^44^. Figure 2d shows the variation of the output Q-factor for bit rates varying from 15 to 45 Gbps and two different RBS lengths of 31 and 127 bits, respectively. As expected, the performance of the NOT gate improves significantly for input signals with a shorter RBS length. The finite amplitude BW (50 GHz) of the phase filter creates a low-pass filtering effect on the incoming data signal, especially for signals with speed exceeding 35 Gbps—leading to a reduction in the resultant Q-factor. System-level simulations using an ideal phase filter validate the observed trend in performance (refer to Fig. 2d). Receiver sensitivity measurements for both the input and output were carried out by varying the average power to the receiver (optical sampling oscilloscope in this case), assuming a 45-Gbps NRZ-OOK input signal with an RBS of 127 bits, results shown in Fig. 2e. As expected, both the input and output-inverted signals follow a near-linear dependence as a function of the average power impinging on the receiver, while the power penalty of the device is ~1 dB for a Q-factor of 3. We should stress that, unlike electronic transistors-based logic gates, the power consumption of the proposed spectral phase-filtering based passive NOT gate does not scale proportionally with the bit rate, instead it remains independent of the bit rate and is exclusively determined by the insertion loss of the FBG (−3.5 dB for the fabricated device). The estimated energy consumption per bit of the device considering 45-Gbps operation is ~34 fJ/bit. Refer to the Methods section for the detailed calculation of the energy consumption metric. In the reported experiments, the energy consumption of the demonstrated passive logic gates is mainly limited by the detection power threshold (or sensitivity) of the available photoreceiver. A more sensitive photoreceiver would require notably lower average power for signal detection (generally, < − 15 dBm) — enabling sub-fJ/bit operation of the proposed linear passive NOT logic.

### Experimental realization of XNOR logic operation

**Fig. 3:**
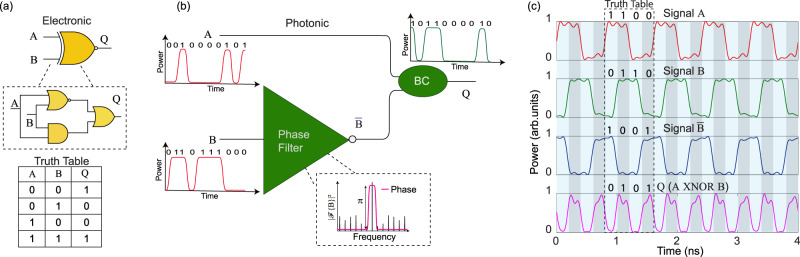
Experimental demonstration of XNOR logic based on spectral phase filtering. **a** The simplest implementation of a XNOR logic in electronics require three mixed logic gates, viz., NOR, AND, and OR. The truth table represents the XNOR output, Q of the binary inputs A and B. **b** The proposed photonic equivalent XNOR logic, which can be realized using a beam combiner (BC) and a phase filter with a single *π* − phase shift. Thus, a distinctly simple, all-fibre implementation can be realized using the FBG-based phase filter device. $\overline{{{{{{{{\rm{B}}}}}}}}}$ denotes the inversion of signal B. **c** Measured temporal waveforms corresponding to the inputs and the resultant output for a simple 4-bit sequence. Through a proof-of-concept experiment, we show that the proposed all-fibre implementation indeed follows the Boolean truth table of a XNOR gate. An input optical NRZ-OOK data sequence at 10.25 Gbps is split and delayed by 1 bit to generate two input data sequences: A and B. The 4-bit sequence is optically generated at a bit rate of 10.25 Gbps (speed of the input signal limited by the acquisition bandwidth of the real-time oscilloscope) using a 10-GHz intensity modulator driven by a 24-GSa/s arbitrary waveform generator (AWG). Signal B is sent to the phase filter using an optical circulator. The inverted signal is then combined with signal A using a beam combiner. The output signal is detected with a 10-GHz photodetector and captured using an 8-GHz real-time scope. Each coloured vertical segment indicates a 1-bit period.

XNOR, or the so-called equivalence gate, is utilized to realize a diverse set of processing functionalities, including error correction/detection^45–48^, cryptography^49^, pattern matching^50^, signal processing^51^, and label-swapping^52^, to name a few. In electronics, the simplest implementation of an XNOR logic gate requires three mixed logic gates, as shown with a schematic representation in Fig. 3a. On the other hand, its photonic equivalent can be realized using only a phase filter and a beam combiner, see Fig. 3b. The XNOR logic operation based on an all-passive frequency domain NOT operation was first theoretically proposed in ref. ^16^, though not experimentally demonstrated. Briefly, a beam combiner sums the two input data signals, where the carrier frequency of one of the input signals gets inverted by the phase filter. At the output of the beam combiner, the constant carrier frequency components of the two signals cancel out, in such a way that the field amplitude output is determined by the sum of the amplitude of the two input data sequences. Thus, the optical output at the combiner consists of three levels in amplitude ( − 2, 0, and 2) or two levels (0 and 1) in intensity, which mimics the logical XNOR operation, as per the Boolean truth table shown in Fig. 3a. We demonstrate, through proof-of-concept experiments, a fully passive XNOR logic operation. We consider a simple case of a 4-bit sequence {1 1 0 0} at 10.25 Gbps, which is split and delayed by 1-bit to create two input sequences: A and B (see Fig. 3c). As shown in Fig. 3c, a high output ‘1’ is obtained if and only if both of the inputs are the same, otherwise, the logic gate results in a low output ‘0’ (refer to the XNOR’s truth table in Fig. 3a). A detailed description of the experimental scheme is provided in the Methods section and the Supplementary information (Supplementary Fig. 2). The estimated energy consumption of the demonstrated XNOR logic considering 10.25-Gbps operation is ~425 fJ/bit. See the Methods section for the detailed calculation. In order to realize a perfect XNOR logic operation (i.e., with complete extinction between the ‘1’ and ‘0’ levels), it is important to employ highly coherent input data signals with identical amplitude levels.

## Discussion

**Fig. 4:**
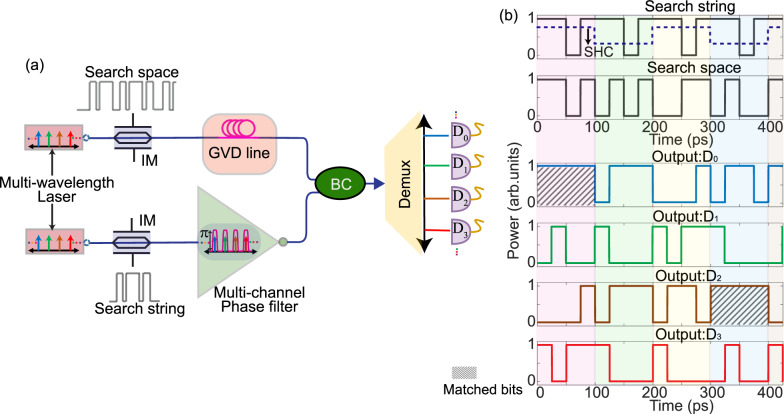
Proposal for a multichannel FBG-based pattern matching algorithm. **a** Schematic illustrating the operational principle wherein the demonstrated fully passive XNOR logic can be extended to realize an all-photonic pattern matching algorithm by employing a multi-wavelength laser, a group-velocity dispersive (GVD) line (providing a 1-bit wavelength dependent shift), and a multi-channel FBG-based phase filter. IM: intensity modulator. Demux: Wavelength-division demultiplexer. **b** Numerical simulations representing pattern matching of a 4-bit search string: {1 1 0 1} within a 16-bit search space: {1 1 0 1 0 1 0 1 0 0 1 1 0 1 0 0}. Notice that the search string is programmed to repeat twice within the search space. Bit sequences are encoded in the NRZ-OOK format at a bit rate of 40 Gbps. Photodetector signals (D_*i*_) correspond to the XNOR output of the *i*^th^− bit shifted search space and the search string, *i* = {0, 1, . . . *N* − 1}. The position of the first bit of the sequence (within the search space) that matches with the search string is given by: *j* = (*Q* − 1) ⋅ *N* − (*i* − 1), where *Q* is the segment number, *Q* = {1, 2, . . . *M*/*N*}, *M* and *N* are the length of the search space and search string respectively. The timing information of individual segments can be determined by utilizing a sub-harmonic clock (SHC), depicted with dashed blue lines. As seen, outputs D_0_, D_2_ have a continuous high or `1’ level over the 4-bit duration of the search string in the 1st and 4th segments respectively (highlighted with hatched lines)—indicating that the search string is present in the search space at two different locations: the 1st and the 11th. Each coloured vertical segment highlights the period of the search string.

The strikingly simple design of passive XNOR logic can be exploited to realize several interesting photonic-based advanced computing applications. For instance, we propose here the use of this passive logic to implement a pattern matching technique offering a potential performance well beyond the reach of present methods, in terms of both processing speed and energy efficiency. Pattern matching involves scanning a large sequence, text, or database to detect locations of a user-defined pattern. Pattern or string matching finds application in a wide range of fields, such as DNA sequence analysis, natural language processing, agricultural research, among many others^53^. However, pattern matching techniques based on state-of-the-art search algorithms in computers remain computationally expensive. For instance, such algorithms can take up to 40 hours of processing time to find locations of a short pattern (only few bits long) within a large database, e.g., as commonly encountered in the field of computational bioinformatics for Genome sequencing tasks^53,54^. Optical pattern recognition techniques include the utilization of coherent multi-line interferometers^55^, matched filters such as VanderLugt (VL) correlators^56^, etc. Such methods can either recognize only a limited number of combinations of an *N*-bit search pattern^55^ or require reprogramming the impulse response of the matched filter either in the time-domain^57^ or along the frequency spectrum domain^58^. To this end, we propose an application of the demonstrated XNOR logic to realize pattern matching by use of a multichannel FBG-based phase filtering device. By simply introducing a phase-only sampling function in the grating equation (Eqn. (1)), it is possible to replicate the single-channel response of the FBG to successive multiple channels, with a desired channel spacing^59^. It is worth mentioning that the device length and the implementation complexity, in terms of the required apodization profile of the resultant phase-sampled FBG, remains identical to the single-channel FBG-device. The basic operation principle of the proposed scheme is as follows (see Fig. 4): first, both the *N*-bit search string (target pattern) and the *M*-bit search space (incoming long sequence) are optically mapped over *N*-successive wavelengths (with a given wavelength-spacing), derived either from a multi-wavelength laser source or a frequency comb. In practice, the length of the search space (*M*) is typically much longer than that of the target pattern (*N*). Then, the optically encoded *M*-bit search space is propagated through a dispersive medium, such as a standard single-mode fibre or a linearly chirped FBG, to introduce a 1-bit wavelength dependent group delay over the *N*-wavelength channels. On the other arm of the beam combiner, the multichannel phase filter is used to invert the *N*-bit search string mapped across multiple channels. Thus, a simultaneous inversion of the *N*-bit search string present in each of the *N*-wavelength channels is realized. At the output of the beam combiner, a demultiplexer followed by *N* photodetectors provide the XNOR output between the *N*-bit search string and the respective bit-shifted *M*-bit search space. A continuous high or ‘1’ level over the *N*-bit duration in any of the detected signals indicate the presence of the search string within the search space. Further, a low-pass RF filter can be employed to reduce the faster oscillations corresponding to the individual bit-by-bit transitions while preserving the relatively slower continuous high level at the output. A schematic of the proposed high-speed photonic-based word-search algorithm with numerical simulations illustrating the concept for a simple case of (*M*, *N*) = (16, 4) is presented in Fig. 4.

Thus, in contrast to previous photonic-based techniques, by exploiting the multichannel response of the FBG, it is possible to realize the standard shift-and-compare functionality of a linear word-search algorithm in a concurrent fashion without a priori knowledge about the target pattern itself. The worst-case complexity of the proposed algorithm is *O*(*n*), where *n* is the number of bits in the target pattern. In contrast to computer-based search algorithms, the processing time of the proposed photonic based pattern matching solution does not scale exponentially with the length of the target pattern, and as such, it can provide orders of magnitude improvement in processing time (at most few seconds compared to 10s of hours), especially in detecting locations of short patterns (e.g., <10 bits) within a large database.

In conclusion, we report the design and realization of a customized FBG-based phase filter with an unprecedented frequency resolution of 1 GHz, at least one order of magnitude improvement compared to an optical waveshaper and previous fibre-optics or integrated phase filter designs. A phase-modulation apodization technique coupled with a femtosecond laser based plane-by-plane direct-writing scheme have been employed to precisely control and define the required coupling coefficient profile along a 21-cm long FBG-device. The remarkable frequency resolution of the FBG has been exploited to realize high-speed, fully passive NOT and XNOR logic operations in an all-fibre compact implementation. Specifically, a photonic-NOT gate operating at 45 Gbps with an estimated energy consumption of ~34 fJ/bit has been experimentally demonstrated by selectively imparting a *π* − phase shift on the carrier frequency component of the input NRZ-OOK signal through the phase filter. The excellent frequency resolution offered by the FBG is the key in enabling operation of the logic gate over a wide range of bit rates, demonstrated here from 15 to 45 Gbps. Moreover, the phase filter has been utilized to experimentally demonstrate a fully passive XNOR logic at a speed of 10.25 Gbps with an estimated energy consumption of ~425 fJ/bit.

The phase-modulation based design framework described in this paper has been recently adopted to synthesize on-chip discrete phase filters for dispersion compensation of both periodic^60^ and aperiodic (e.g., telecom data) signals^61^ using waveguide Bragg gratings (WBGs) in a silicon on insulator (SOI) platform. By implementing the phase-only filter using WBGs designed in a spiral geometry^62^, several orders of magnitude reduction in the overall device footprint, i.e., from few cm^2^ to sub-mm^2^, could be realized. Towards circulator-free operation of reflective devices, a myriad of alternative solutions have been proposed to collect the reflected signal from Bragg gratings, such as contra-directional couplers^63^, asymmetric Y-splitters^64^, mode-converters assisted coupling elements^65^, etc. We anticipate that such coupling elements could be employed to cascade multiple WBG-based spectral phase filters to realize sophisticated logic operations. Further, the resistive-heating scheme developed in this work for the localized correction of phase errors could be easily adapted to on-chip integrated platforms, by fabricating metallic thin-film heaters on top of WBGs, potentially allowing for the realization of distortion-free ultra-compact all-pass spectral phase filters. Additionally, we envision the implementation of an optimization algorithm-assisted tuning scheme for the realization of energy-efficient reconfigurable multifunctional phase filtering devices for a host of important signal processing functionalities, such as telecom data format conversion^66^, clock recovery^23^, and many others.

## Methods

### FBG design and fabrication

The implementation of the phase-only filter, *H*(*jω*) = ∣*H*(*jω*)∣*exp*(*j∠H*(*jω*)) is achieved using an FBG operating in reflection. The target phase response (*∠H*(*jω*)) consists of a rectangular 0 − *π* phase shift with a 3-dB bandwidth (BW) of 1 GHz, while the amplitude spectral response ∣*H*(*jω*)∣ exhibits an 8^th^ −order super-Gaussian function shape, with a peak intensity (∣*H*(*jω*)∣^2^) of 0.5 and a 3-dB BW of 50 GHz (see Fig. 1b). To realize a higher target BW and/or peak reflectivity, a large peak coupling coefficient value of the FBG is required, see the Supplementary Information (Supplementary Fig. 1). For the femtosecond laser based direct-writing scheme used in the inscription of the designed FBG, the peak coupling coefficient value that can be realized without introducing additional distortions is ~5 cm^−1^. Hence, we limit the target 3-dB BW and the peak reflectivity values to 50 GHz and 50% respectively. A phase-modulation based apodization scheme^43^ is adopted to achieve the defined stringent phase filtering response required for the realization of the NOT operation on arbitrary data signals. The target apodization is achieved by incorporating a slowly varying sinusoidal phase component, *ϕ*_AP_(*z*), in the phase function of the FBG, where *ϕ*_AP_(*z*) = *ϕ*_0_(*z*) ⋅ *sin*(2*π*/Λ_*P*_(*z*)) is the apodization phase function having a slowly modulating amplitude *ϕ*_0_ and phase-modulation period Λ_*P*_. *ϕ*_0_(*z*) is calculated from the normalized target apodization profile, ∣*κ*_*n*_(*z*)∣, by a 0th − order Bessel function, namely, ${\phi }_{0}(z)={J}_{0}^{-1}(|{\kappa }_{n}(z)|)$. The grating total phase ((2*π*/Λ)*z* + *ϕ*_*κ*_(*z*) + *ϕ*_AP_(*z*)) is used to calculate the separation between each of the grating planes along the FBG. The inscription of this structure is achieved with a femtosecond laser based modified plane-by-plane direct-writing scheme, in which a highly accurate control strategy is implemented to precisely define the position of each grating plane, making it possible to use a phase-modulation period of just a few *μ*m, thus enabling the realization of the complex target apodization function with a very high spatial resolution^37^.

### Experimental setup for NOT gate

A 92-GSa/s arbitrary waveform generator (AWG) [Keysight M8196A] is used to drive the 40-GHz intensity modulator [Optilab] to generate the input data signal at varying bit rates and random bit sequence (RBS) lengths. The resultant signal is amplified and coupled to the FBG using an optical circulator. The temporal waveforms and the corresponding eye diagrams are recorded using a 500-GHz optical sampling oscilloscope (OSO) [EXFO PSO-101]. A variable optical attenuator ensured that the waveforms are recorded at a fixed input power to the OSO. The optical spectrum of the input and output-inverted data signal are measured using an optical spectrum analyzer [Apex 2043B, resolution 20 MHz].

### Experimental setup for XNOR logic

We should note that due to random phase fluctuations between the two arms of the beam combiner, the intensity of the output signal undergoes random oscillations, typically encountered in any fibre-based interferometric configuration. To this end, a secondary CW laser is employed, whose centre wavelength is fixed at an offset of 0.2 nm to the central wavelength of the phase filter. The outputs from the secondary and the primary lasers are then combined and sent to an intensity modulator with a 3-dB bandwidth of 10 GHz driven by a 24-GSa/s AWG [Tektronix 7122C]. An optically modulated 4-bit sequence at 10.25 Gbps speed is split and delayed by 1 bit to generate two input 4-bit data streams (A and B). Signal B is sent to the FBG via an optical circulator. A fibre-based beam combiner is used to combine the output-inverted signal from the FBG and signal B. Any temporal misalignment between the two signals is fixed using an optical delay line. At the output of the beam combiner, a programmable optical waveshaper [Finisar 4000S] is used as a demultiplexer to separate the two wavelengths. The photodetected (non-inverted) signal from the secondary laser is used as a trigger for the scope to capture the temporal output waveforms using the edge-detection technique of the real-time scope [Tektronix MSO70804C]. Thus, by properly adjusting the trigger level of the scope, it is possible to capture the output waveform when the signals in the two arms of the interferometer interfere constructively, as any phase fluctuations-induced variations at the output impact both wavelengths equally. The schematic of the experimental setup is presented in the Supplementary information (Supplementary Fig. 2).

### Energy consumption metric

Since the proposed phase-filtering based logic is entirely passive, the energy consumption per bit metric of the logic gate is determined exclusively from the insertion loss of the phase filter, input power and operational speed. The measured insertion loss (*IL*) of the phase filter is ~3.5 dB, which leads to an estimated energy consumption per bit, *E*_Bit_ = (*P*_IP_ − *P*_OP_) ⋅ Δ*t* = *P*_IP_(1 − 10^−*IL*/10^) ⋅ Δ*t* ~34 fJ/bit, where *P*_IP_ ~2.8 mW (4.5 dBm) and *P*_OP_ ~1.26 mW (1 dBm) are the average power of the input and output signals respectively. Δ*t* ~22.22 ps is the bit period for the demonstrated 45-Gbps operation. Similarly, for the XNOR logic, the *E*_Bit_ is calculated to be ~425 fJ/bit for 10.25 Gbps operation speed, *P*_IP_ ~ 5.6 mW (7.5 dBm) and *P*_OP_ ~ 1.26 mW (1 dBm). Note that the total insertion loss in this case is 6.5 dB (additional 3 dB due to the beam combiner at the output). We should highlight that *P*_OP_ is determined by the sensitivity (or power detection threshold) of the photoreceiver (1 dBm in the present case). A reduced energy consumption could then be achieved by simply using a sensitive photoreceiver, as this would enable reducing the input signal energy. For instance, considering a photoreceiver with a sensitivity of − 15 dBm, the energy consumption of the NOT and XNOR logic gate would be ~0.8 fJ/bit and ~10 fJ/bit, respectively, at the demonstrated operational speeds.

### Condition for distortion-free operation of passive logic gates

To ensure distortion-free operation, the 3-dB BW of the (0 − *π*) phase shift or the frequency resolution (*ν*_*r*_) of the phase-only filter should meet the following condition:

2 $$\Delta {\nu }_{{{{{{{{\rm{cw}}}}}}}}} \, < \, {\nu }_{r} \, < \, 2B/{{{{{{{{\rm{RBS}}}}}}}}}_{{{{{{{{\rm{length}}}}}}}}}$$

where Δ*ν*_cw_ is the linewidth of the continuous-wave (CW) laser and RBS_length_is the length of the input RBS with bit rate *B*. The upper-limit of *ν*_*r*_ is determined by noting that individual discrete frequency components of an RBS are periodic with a spacing given by *B*/RBS_length_. Thus, Eqn. (2) ensures that the *π* − phase shift is selectively applied only over the carrier frequency component of the input signal, since a non-zero phase shift over some of the discrete spectral components of the input data signal would result in undesired additional amplitude modulation between the output bits.

### Reporting summary

Further information on research design is available in the Nature Portfolio Reporting Summary linked to this article.

## Supplementary information

Supplementary Information

Reporting Summary

## Data Availability

The datasets generated during the current study are available from the corresponding author on request.

## Code availability

The codes used in the present work for simulations and data analysis are available from the corresponding author on request.
