# Supplementary material for: All-fibre phase filters with 1-GHz resolution for high-speed passive optical logic processing
Source: Nat Commun. 2023 Mar 31;14:1808. doi: 10.1038/s41467-023-37472-2 (PMC10066316; doi:10.1038/s41467-023-37472-2)
Supplement: Supplementary file 1 — Supplementary Information [file 41467_2023_37472_MOESM1_ESM.pdf]

Supplementary Information

All-fibre phase filters with 1-GHz resolution for high-speed passive optical logic processing

Saket Kaushal[1], A. Aadhi[1], Anthony Roberge[2], Roberto Morandotti[1], Raman Kashyap[2,3] and José Azaña[1*]

[1]Énergie, Matériaux et Télécommunications, Institut National de la Recherche Scientifique, 1650 Lionel-Boulet Blvd., Varennes, J3X 1P7, Quebec, Canada.
[2]Department of Engineering Physics, Fabulas Laboratory, Polytechnique Montréal, 2500 Chem. de Polytechnique, Montréal, H3T 1J4, Quebec, Canada.
[3]Department of Electrical Engineering, Fabulas Laboratory, Polytechnique Montréal, 2500 Chem. de Polytechnique, Montréal, H3T 1J4, Quebec, Canada.

*Corresponding author. E-mail: jose.azana@inrs.ca;

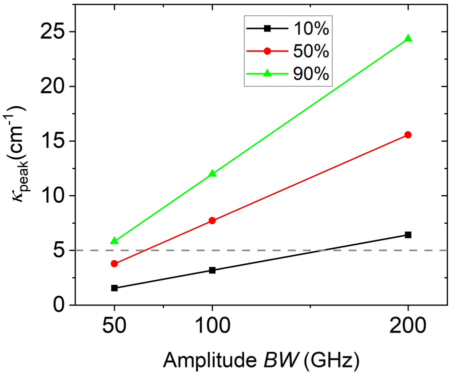

Supplementary Fig. 1 Variation of coupling coefficient peak value (κ_{peak}) with target amplitude bandwidth (BW) and peak reflectivity of the fibre Bragg grating (FBG). The dashed horizontal line represents the upper limit of the coupling coefficient value ($\sim$ 5 cm^{-1}) that can be realized with the femtosecond laser-based plane by plane direct writing scheme.

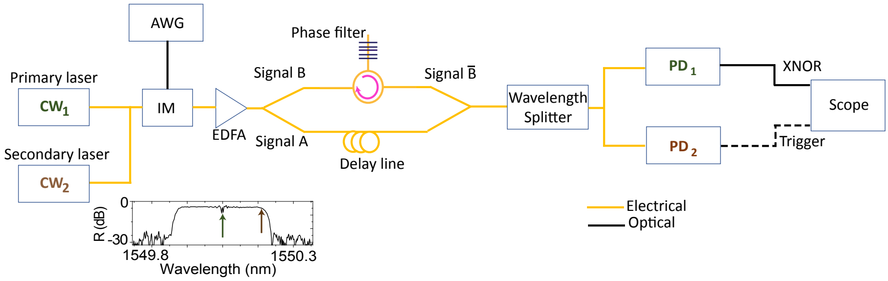

Supplementary Fig. 2 Schematic of the experimental setup for the demonstration of passive XNOR logic using the FBG-based phase filter. CW: continuous wave, IM: intensity modulator, AWG: arbitrary waveform generator, EDFA: erbium-doped fibre amplifier, PD: photodiode
